# Benign paroxysmal positional vertigo after radiologic scanning: a case series

**DOI:** 10.1186/1752-1947-2-92

**Published:** 2008-03-27

**Authors:** Erdinc Aydin, Kubra Akman, Hasan Yerli, Levent N Ozluoglu

**Affiliations:** 1 Baskent University Faculty of Medicine, Department of Otorhinolaryngology, Ankara, Turkey; 2 Baskent University Zubeyde Hanim Practice and Research Center, Department of Radiology, Izmir, Turkey

## Abstract

**Introduction:**

Benign paroxysmal positional vertigo (BPPV) is the most common type of vertigo. It is frequently seen in elderly patients, and the course of the attack may easily mimic cerebrovascular disease. A BPPV attack after a radiologic examination has not been reported previously. We report the cases of two patients who had BPPV attacks after radiologic imaging.

**Case presentation:**

The first patient with headache and tremor was admitted to the radiology department for cranial computed tomography (CT) imaging. During scanning, she was asked to lie in the supine position with no other head movements for approximately 10 minutes. After the cranial CT imaging, she stood up rapidly, and suddenly experienced a vertigo attack and nausea. The second patient was admitted to the radiology department for evaluation of his renal arteries. During the renal magnetic resonance angiography, he was in the supine position for 20 minutes and asked not to move. After the examination, he stood up rapidly with the help of the technician and suddenly experienced a vertigo attack with nausea and vomiting. The results of standard laboratory analyses and their neurologic examinations were within normal limits and Dix-Hallpike tests showed rotatory nystagmus in both cases. An Epley maneuver was performed to the patients. The results of a control Dix-Hallpike tests after 1 Epley maneuver were negative in both patients.

**Conclusion:**

Radiologists and clinicians must keep in mind that after radiologic imaging in which the patient is still for some time in the supine position and then helped to stand up rapidly, a BPPV attack may occur.

## Introduction

Benign paroxysmal positional vertigo (BPPV) is the most common type of vertigo and is defined as a vestibular syndrome of peripheral origin characterized by short and intense episodes of vertigo, associated with predominantly horizontal-rotation nystagmus, triggered by a quick change of head position [1-4]. It is benign because it is not progressive; paroxysmal because it is sudden and unpredictable in onset; positional because it comes about because of a change in head position; and vertigo because of a sense of spinning of the room or whirling. It is more often seen in women than in men [5]. It occurs at all ages, but its occurrence increases with age. Long-term bed rest, extended travel, head trauma, and upper respiratory system infections are believed to predispose patients to vertigo attacks [1-5]. Many patients also have vegetative symptoms such as nausea or vomiting and balance disorders.

The clinical pathology substrate corresponding to BPPV was proposed in 1962 by Schuknecht, who described the presence of crystals coming from the utriculus macula, which are released and then adhere to the top of the posterior semicircular canal [4]. Since its first description by Dix and Hallpike, the maneuver named after them has become the standard test for diagnosing BPPV [2,6]. In this test, the patient's head is rotated 45° toward the side to be tested. Then the patient is suddenly moved to the supine position with the head hyperextended approximately 20° [6]. The nystagmus observed in BPPV has a latency of three to 30 seconds and persists for a few seconds [6]. Advocated treatments are maneuvers of canalith repositioning (the Epley maneuver is the most common one) and surgical treatments, such as singular neurectomy. Some investigators have reported variations of the Epley maneuver [6]. Although the Epley maneuver and the augmented Epley maneuver have been found to be effective, the augmented Epley maneuver is considered as being better tolerated by obese patients and in patients with musculoskeletal disorders [7]. In the simpler version of the maneuver, after a Dix-Hallpike test, the patient's head is rotated 90° toward the unaffected side and then the patient sits up. In the augmented version however, after the Dix-Hallpike test, the patient's head is rotated 90° toward the unaffected side and later, is asked to roll about the vertical axis toward the same side before sitting up [7]. Regardless of the type of repositioning maneuver, treatment is effective in 70% to 90% of cases, and this percentage is not affected by the age of the patient. However, previous studies have shown that more than one maneuver may be needed to decrease vertigo or spontaneous nystagmus on the Dix-Hallpike test [5].

A BPPV attack after a radiologic examination has not been reported previously. We report the cases of two patients who had BPPV attacks after radiologic imaging. They both were elderly patients, and both were asked to remain in the supine position for some time. They both experienced the vertigo attack right after scanning, while standing up. In both cases, radiologists suspected cerebrovascular disease.

## Case presentation 1

The first patient was a 66-year-old woman with comorbid osteoporosis, osteoarthritis, hyperlipidemia, and hypertension. She had gone to the physical therapy and rehabilitation department with tremor in her hands and legs, and headache. The clinician referred the patient to the radiology department for cranial computed tomography (CT) imaging. During scanning, she was asked to lie in the supine position with no other head movements for approximately 10 minutes. After the cranial CT imaging, she stood up rapidly, and suddenly experienced a vertigo attack and nausea. We referred the patient to the emergency department. The results of standard laboratory analyses and her neurologic examination were within normal limits, and no spontaneous nystagmus was observed. A Dix-Hallpike test was performed and during the right-side swing, the patient experienced vertigo and rotatory nystagmus was observed. The nystagmus had 4 to 7 seconds of latency and lasted for approximately 20 seconds. The Epley maneuver was performed to the right side, and she was sent home. After the first Epley maneuver, the patient was asked to revisit our clinic 3 days later. The results of a control Dix-Hallpike test were negative, and the patient was symptom-free 3 days later.

## Case presentation 2

The second patient was a 72-year-old man with a history of diabetes mellitus, hypertension, hyperlipidemia, and coronary artery disease. Owing to his elevated creatinine and blood urea nitrogen levels, the internal medicine department planned renal magnetic resonance angiography scanning. During the procedure, he was in the supine position for 20 minutes and asked not to move. After the renal magnetic resonance angiography, he stood up rapidly with the help of the technician and suddenly experienced a vertigo attack with nausea and vomiting. Similar to the first patient, we referred him to the emergency department. The results of a head and neck examination were within normal limits, and no spontaneous nystagmus was observed. A Dix-Hallpike test was performed. During the left swing, the patient had severe vertigo, and rotatory nystagmus started after 3 to 6 seconds and was observed for 25 to 30 seconds. An Epley maneuver was performed to the left side. The results of a control Dix-Hallpike test after 1 Epley maneuver were negative, and he was symptom-free 3 days later.

In both cases, the patients did not need an additional Epley maneuver because they experienced no further vertiginous symptoms.

## Discussion

The peripheral vestibular system is located in the inner ear and is composed of a bony labyrinth, a membranous labyrinth, and specialized hair cells responsible for detecting motion. Within each temporal bone of the skull is the bony labyrinth, which contains 3 semicircular canals and a central chamber called the vestibule. The bony labyrinth is filled with perilymphatic fluid in which the membranous labyrinth is suspended. Situated inside the membranous labyrinth are the 5 sensory organs of the peripheral vestibular system: the 2 otoliths (the utricle and saccule) and the 3 semicircular canals connected to the vestibule. The hair cells within each of the sensory organs are responsible for converting mechanical information from head motions into neural signals.

The movement of fluid within these canals allows the brain to sense rotation of the head through all 3 directions in space. The primary function of the semicircular canals is to sense angular acceleration of the head. The saccule and utricle are responsible for detecting linear acceleration of the head. The otoliths are also sensitive to tilts of the head with respect to gravity.

The hair cell is the basic sensory element of the peripheral sensory apparatus. Present in the saccule, utricle, and the cristae of the semicircular canals, these hair cells transduce mechanical force into electrical nerve action potentials. Each hair cell is innervated by an afferent neuron, and each hair cell has a large number of small cilia. A gelatinous membrane (called the cupula in the semicircular canals) overlies each set of hair cells. In the otoliths, calcium carbonate crystals (called otoconia) are located within the gelatinous membrane resting on top of the hair cells. This makes the otoliths gravity-sensitive, an attribute not shared by the semicircular canals.

Otoconia can become mechanically dislodged from the utricle and may enter into the cupula of one of the semicircular canals (cupulolithiasis) or float around in the endolymph (canalithiasis). These small calcium carbonate crystals that float through the inner ear fluid strike against sensitive nerve endings (the cupula) within the balance apparatus at the end of each semicircular canal (the ampulla) and produce position- or motion-induced vertigo and disequilibrium, which is referred to as BPPV [3]. In elderly patients, otoconia are frequently lodged in the semicircular canals. Therefore, during sudden head movements, these otoconia float around and irritate the hairy cells, causing vertigo. We believe that the mechanism of vertigo in our patients was elicited by the position of the patients, not the radiologic tests.

## Conclusion

BPPV has a good prognosis, but it is a challenging disease. It is frequently seen in elderly patients, and the course of the attack may easily mimic cerebrovascular disease. Thus, radiologists and clinicians must bear in mind that after radiologic imaging in which the patient is still for some time in the supine position and then helped to stand up rapidly, a BPPV attack may occur.

## Competing interests

The author(s) declare that they have no competing interests.

## Authors' contributions

EA and KA wrote the first draft of the manuscript. EA obtained patient consent. KA and HY performed the literature search. HY and LO revised the first draft of the manuscript for submission. All authors read and approved the final manuscript.

## Consent

Written consent was obtained from both patients for publication of this case report. A copy of the written consent is available for review by the Editor-in-Chief of this journal.
